# The production of wax esters in transgenic plants: towards a sustainable source of bio-lubricants

**DOI:** 10.1093/jxb/erac046

**Published:** 2022-02-10

**Authors:** Frédéric Domergue, Magdalena Miklaszewska

**Affiliations:** 1 Univ. Bordeaux, CNRS, LBM, UMR 5200, F-33140 Villenave d’Ornon, France; 2 Department of Functional and Evolutionary Ecology, Division of Molecular Systems Biology (MOSYS), Faculty of Life Sciences, University of Vienna, Djerassiplatz 1, 1030, Vienna, Austria; 3 Department of Plant Physiology and Biotechnology, University of Gdańsk, Wita Stwosza 59, 80-308, Gdańsk, Poland; 4 Sogang University, South Korea

**Keywords:** Fatty acyl reductase, green factory, lubricant, oilseed crops, plant metabolic engineering, wax esters, wax synthase

## Abstract

Wax esters are high-value compounds used as feedstocks for the production of lubricants, pharmaceuticals, and cosmetics. Currently, they are produced mostly from fossil reserves using chemical synthesis, but this cannot meet increasing demand and has a negative environmental impact. Natural wax esters are also obtained from *Simmondsia chinensi*s (jojoba) but comparably in very low amounts and expensively. Therefore, metabolic engineering of plants, especially of the seed storage lipid metabolism of oil crops, represents an attractive strategy for renewable, sustainable, and environmentally friendly production of wax esters tailored to industrial applications. Utilization of wax ester-synthesizing enzymes with defined specificities and modulation of the acyl-CoA pools by various genetic engineering approaches can lead to obtaining wax esters with desired compositions and properties. However, obtaining high amounts of wax esters is still challenging due to their negative impact on seed germination and yield. In this review, we describe recent progress in establishing non-food-plant platforms for wax ester production and discuss their advantages and limitations as well as future prospects.

## Teaser

We review the most recent developments in metabolic engineering of plants towards the production of wax esters for industrial applications.

## Introduction

**Fig. 1.**
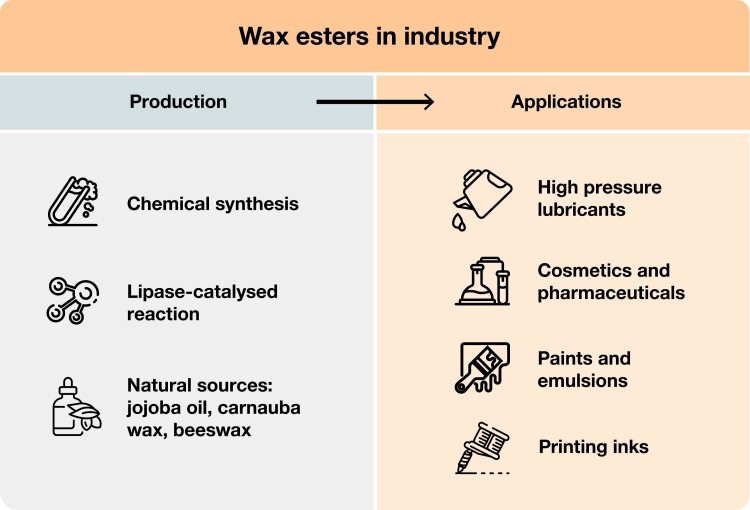
Wax ester production methods and industrial applications. Wax esters are important industrial lipids used as ingredients for the formulation of lubricants, cosmetics, pharmaceuticals, paints, emulsions, and printing inks. They can be produced by chemical synthesis or lipase-catalysed esterification of petroleum products or plant oils. Nowadays, natural wax esters are mainly obtained from jojoba oil, carnauba wax and beeswax.

Wax esters (WEs) are a class of neutral lipids composed of fatty acids esterified with fatty alcohols. They are widely used in different industrial sectors to produce surface coatings, polishes, printing inks, and candles, as well as for cosmetic and pharmaceutical applications (Fig. 1). In particular, WEs with a low melting point and a high oxidation stability are highly in demand as components of lubricants (Carlsson *et al.*, 2011). In the past, WEs for lubrication purposes were obtained from the sperm whale (*Physeter macrocephalus*), which can accumulate up to 4 tons of this valuable oil in its skull (Clarke, 1979). As a consequence, the sperm whale became almost extinct and was classified as an endangered species in the early 1970s. The ensuing ban on sperm whale hunting and the import of whale products resulted in an active search for suitable resources for replacing spermaceti oil (Nieschlag *et al.*, 1977). The desert plant jojoba (*Simmondsia chinensis*), which accumulates WEs instead of triacylglycerols (TAGs) as storage lipids (Miwa, 1971), was considered as a potential substitute. Nevertheless, jojoba is a low yield plant that can only be cultivated on a limited scale in hot and dry climates. Thus, jojoba oil remains expensive and its use is restricted to the cosmetic sector (Carlsson *et al.*, 2011). Nowadays, most WEs are produced through complex chemical processes relying on high energy consumption and fossil fuel resources. In order to reduce dependence on petroleum and to decrease the environmental impact of WE production, the development of so-called green factories for the production of bio-based lubricants represents a very promising alternative that would combine both sustainability and improved biodegradability. This review describes the different strategies that have been developed to produce WEs in plants, the first promising results, as well as possible ways to improve the quantity and quality of the desired end-products.

## Wax esters: chemical structure and functions

Although WEs are simply composed of two acyl-chains, a great degree of WE structural diversity exists in nature (see Box 1 for WE nomenclature). While most WEs consist of even-chain saturated and unsaturated fatty acyl and alcohols moieties ranging from C12 to C30 carbons in length (Patel *et al.*, 2001), WEs containing odd-chain or branched components, isoprenoid alcohols or diols have also been described (Holtzapple and Schmidt-Dannert, 2007; Chinta *et al.*, 2016). WEs have been found in organisms from all kingdoms of life (except Archae and fungi) and shown to fulfill several important biological functions. Most commonly, WEs are found in surface lipid layers: as constituents of the cuticle of plants (Li *et al.*, 2008) and the exoskeleton of insects (Nelson *et al.*, 2001), and as secretions produced by sebaceous glands in mammalian skin (Cheng and Russell, 2004a), they protect from water loss, pathogen attack, and ultraviolet light. In birds, WEs are produced by the uropygial gland for the lubrication, waterproofing, and maintenance of the plumage (Biester *et al.*, 2012b). WEs also serve as energy storage in bacteria belonging to the genera *Acinetobacter*, *Marinobacter*, *Rhodococcus*, and *Mycobacterium*, in some zooplankton organisms (Lee *et al.*, 2006), and in the phytoflagellate *Euglena gracilis*, which has the ability to synthesize WEs under anaerobic conditions (Inui *et al.*, 1982; Teerawanichpan and Qiu, 2010). Interestingly, the plant kingdom also contains a WE-storing species, *S. chinensis* (jojoba), a desert shrub native to North America, which accumulates WEs in its seeds to sustain post-germinative growth (Miwa, 1971; Sturtevant *et al.*, 2020). Other functions of WEs include buoyancy regulation in marine organisms, such as the sperm whale and copepods (Clarke, 1978; Pond and Tarling, 2011), and chemical communication in insects and birds (Chinta *et al.*, 2016; Grieves *et al.*, 2019).

Among naturally abundant WE sources, the spermaceti oil is a mixture of TAGs and C24–42 WEs, the latter accounting for more than 70% of the total (Nevenzel, 1970). Highly heterogeneous WE compositions within the spermaceti organ have been reported (Morris, 1975) and as many as 240 different WEs were detected by Spencer (1979). The exact WE composition seems to be highly dependent on the specimen (its age and diet) and may not be systematically dominated by cetyl palmitate as often believed (Horiguchi *et al.*, 1999). As mentioned above, this source of WEs is no longer available in order to protect sperm whales. The well-known jojoba oil, widely used in the cosmetic and pharmaceutical industries (Sánchez *et al.*, 2016), is mainly composed of very long-chain monounsaturated (20:1, 22:1, and 24:1) fatty acids and alcohols (Miwa, 1971). Different studies have shown that performance of jojoba oil as a lubricant is similar or even superior to that of spermaceti oil (Gisser *et al.*, 1975; Miwa *et al.*, 1979). However, due to the rather high melting point (around 9 ºC), its usage as a lubricant in cold climates is limited (Carlsson *et al.*, 2011). WEs are also obtained from the epicuticular waxes covering the leaves of the Brazilian palm tree *Copernicia cerifera* (carnauba wax), or the stems of the bush *Euphorbia cerifera* (candelilla wax). According to Doan *et al.* (2017), carnauba wax consists of 62% wax esters, which are composed of C16–C24 fatty acids and C18, C30, and C32 fatty alcohols, while candelilla wax contains only 16% WEs with C16 fatty acids and C18 and C30 fatty alcohols as predominant constituents. WEs are also a major component of beeswax (58%), which is extracted from honey combs. Beeswax mainly comprises C16 fatty acids and C24–C32 fatty alcohols (Doan *et al.*, 2017; Moreau *et al.*, 2018). Other sources of WEs include sheep wool max (lanolin), sorghum kernels, sunflower oil, and rice ban oil. Natural WEs are used as ingredients in cosmetics, food products, polishes, and coating agents (Harron *et al.*, 2017; Moreau *et al.*, 2018).

## Properties of wax esters and industrial applications

The properties of WEs depend on the chemical structure of their fatty acid and alcohol components (Patel *et al.*, 2001). The two key factors influencing WE utility for industrial applications are the melting point and resistance to oxidation, but the thermal and pressure stability are also important. The melting temperature (*T*_m_) is determined by the carbon chain length, the degree of unsaturation, and the position(s) of the double bond(s). It was shown that for synthetic saturated WEs, the *T*_m_ increased by 1–2 °C per additional carbon unit, from approximately 38 °C for a C26 WE (dodecyl myristate, 12:0–14:0) to 75 °C for a C48 WE (tetracosanyl tetracosanate, 24:0–24:0). For WE isomers, the *T*_m_ is affected by the position of the ester linkage, with symmetrical WEs having the highest melting point. Moving the ester bond towards either end of the molecule results in decreasing the *T*_m_ by 1–5 °C (Patel *et al.*, 2001). Saturated, monoenoic, and dienoic WEs of the same length have a very different *T*_m_ (Iyengar and Schlenk, 1969; Patel *et al.*, 2001). For example, the presence of one or two double bonds in WEs composed of C18 moieties decreases the *T*_m_ by approximately 30 °C and 60 °C, respectively (Patel *et al.*, 2001). Moreover, Iyengar and Schlenk (1969) observed that the *T*_m_ of various monounsaturated WEs with the double bond in the fatty acid chain is 10 °C lower in comparison with their isomers with the double bond in the fatty alcohol moiety. Similarly, the *T*_m_ determined by Russell and Volkman (1980) for stearyl palmitoleate (18:0–16:1) was lower than that of oleyl palmitate (18:1–16:0), but with a difference of only 1.5 °C. Oxidation stability is mainly correlated with the degree of unsaturation of a lipid molecule. WEs with a higher number of double bonds are therefore more susceptible to oxidation, which can lead to their polymerization and degradation (Kodali, 2002; Fox and Stachowiak, 2007). In contrast, the presence of branched chains lowers the melting point of WEs (Patel *et al.*, 2001) without negatively affecting oxidative stability.

WEs have been used for decades in many different sectors (Fig. 1). Whereas solid WEs are common ingredients of candles and polishes, liquid WEs are often found in printing inks, paints, surface coatings, leather waterproofing treatments, plasticizers, and oil solutions for lamps. WEs have also many applications in the food, pharmaceutical, and cosmetic sectors. For example, cetyl octanoate (16:0–8:0) is used in the formulation of different cosmetics, including cleansing products, hair conditioners and makeup removers due to its ability to retain moisture (‘Final report on the safety assessment of cetearyl octanoate’, 1982; Kuo *et al.*, 2012b). Nevertheless, in these application sectors, natural sources of WEs are preferred as feedstocks. The very high costs and low yields associated with culturing *S. chinensis* resulted in using jojoba oil only for dermatological formulations, health care products, and cosmetics. Finally, WEs with high oxidative stability and resistance to hydrolysis have outstanding lubrication properties. Such WEs, therefore, represent excellent components of high-performance factory machine lubricants and automobile transmission fluids (Carlsson, 2006). The sulfurized form of spermaceti oil was indeed considered as an ideal additive in many lubricant applications until sperm whale hunting was banned (Nieschlag *et al.*, 1977).

## Current large-scale wax ester production methods

Nowadays, large-scale production of WEs for industrial applications is based on chemical processes using petroleum or plant resources as feedstocks. Alternatively, WEs can be generated by enzymatic synthesis using lipases (Fig. 1). The chemical synthesis of WEs first requires the reduction of a fatty acid to an alcohol, followed by esterification with a fatty acid. Although seemingly simple, this multi-step process is rather expensive, generates waste, and requires high temperatures and pressure, catalysts such as sulfuric acid, tin or titanium, and a complex downstream purification (Lokotsch *et al.*, 1996; Nguyen *et al.*, 2017). Nevertheless, chemical esterification of fatty alcohols with fatty acids enables the production of synthetic WEs with properties similar to spermaceti oil or other natural WEs (Bell *et al.*, 1977; Nieschlag *et al.*, 1977). Lipase-catalysed synthesis, which can be carried out under moderate temperature and pressure conditions in solvent-free systems, has received increasing attention as an attractive alternative to chemical synthesis. The process has lower energy consumption and generates less waste compared with chemical methods (Petersson *et al.*, 2005). In the presence of saturating concentrations of fatty alcohols and under low water content conditions to avoid the reverse reaction, lipases can catalyse alcoholysis/transesterification of oil (TAGs) or esterification of free fatty acids (Vilas Bôas and Castro, 2022). Among a large number of tested WE-synthesizing lipases, enzymes from *Candida*, *Rhizopus*, and *Rhizomucor* species, including the commercially available Lipozyme RMIM (lipase from *R. miehei*) and Novozym 435 (lipase from *C. antarctica*), efficiently produced different long-chain esters resembling natural WEs (Steinke *et al.*, 2001; Guncheva and Zhiryakova, 2008; Lopes *et al.*, 2011; Kuo *et al.*, 2012a; Ungcharoenwiwat *et al.*, 2016). Immobilization of lipases allows the reuse of the enzymes and improves their activity and stability (Kuo *et al.*, 2012a). However, despite many advantages, lipase-based synthesis of WEs is still not productive enough to compete with chemical synthesis (Nguyen *et al.*, 2017). It should also be noted that even though lipase-catalysed production of WEs is more environmentally friendly than the conventional chemical process, it still requires fatty alcohols, which are most commonly produced by hydrogenation of plant and animal oils or from petrochemical feedstocks using the Ziegler process or oxo synthesis (Noweck and Grafahrend, 2006). Both methods suffer from harsh conditions, hazardous reagents, and production of chemical waste (Hagström *et al.*, 2013; Munkajohnpong *et al.*, 2020).

As pointed out above, there is no readily available source of WEs since the ban on the hunting of spermaceti whales, and current methods for obtaining large amounts of WEs for industrial purposes are not only harmful for the environment but also dependent on diminishing fossil reserves. In coming years, WE production may be limited by growing costs, and thus insufficient to meet the increasing demand. Therefore, there is a strong need for alternative bio-based methods for sustainable production of WEs. Metabolic engineering has enabled the establishment of WE synthesis in bacteria (Kalscheuer *et al.*, 2006), yeast (Kalscheuer *et al.*, 2004; Wenning *et al.*, 2019), and plants (Heilmann *et al.*, 2012; Iven *et al.*, 2016; Zhu *et al.*, 2016; Ruiz-Lopez *et al.*, 2017). Among these WE-producing platforms, plants represent an attractive strategy for renewable, sustainable, and environmentally friendly synthesis of WEs tailored to industrial applications.

## Enzymes involved in synthesis of wax esters

**Fig. 2.**
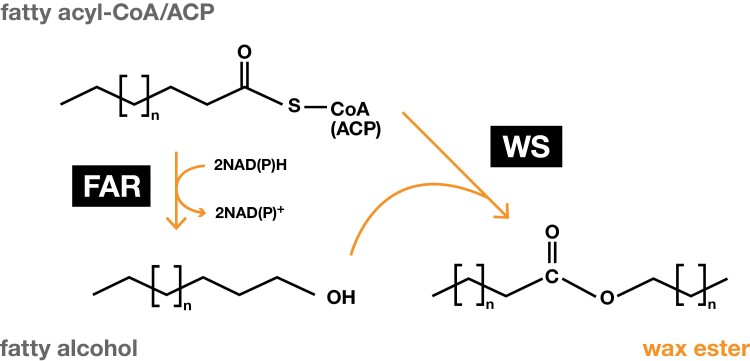
Wax ester biosynthesis. The wax ester biosynthetic pathway involves two steps. First, NAD(P)H-dependent reduction of an acyl-CoA/ACP to the corresponding alcohol is catalysed by a fatty acyl-CoA reductase (FAR). Next, a wax synthase (WS) esterifies an acyl-CoA/ACP with a fatty alcohol to produce a wax ester. Free coenzyme A (CoA) or acyl carrier protein (ACP) is released in both reactions.

The biosynthesis of WEs from acyl-chains is straightforward as it relies on only two consecutive enzymatic activities. First, a fatty acyl reductase (FAR) reduces an acyl-chain to the corresponding fatty alcohol. Next, a wax ester synthase (WS) transfers the acyl group of an acyl-CoA onto the hydroxyl group of the fatty alcohol, yielding a WE (Fig. 2). This pathway was first described in the early 2000s in jojoba (Lardizabal *et al.*, 2000; Metz *et al.*, 2000), and afterwards in many species from bacteria to protists, birds, and mammals.

### Fatty acyl reductases

FARs are acyl-thioester reductases (EC 1.2.1.50). They use NAD(P)H as reducing equivalents, and usually generate fatty alcohols from activated acyl-chains (Rowland and Domergue, 2012). However, some FARs from bacteria and cyanobacteria also produce fatty aldehydes (Reiser and Somerville, 1997; Lin *et al.*, 2013). Pioneering biochemical studies on cell free preparations of etiolated *Euglena gracilis* cells showed that the fatty alcohol production occurred via an aldehyde intermediate that was not released by the FAR enzyme. Several early studies showed that this activity was associated with the microsomal fraction, and used acyl-CoAs as substrates (Kolattukudy, 1970; Pollard *et al.*, 1979; Vioque and Kolattukudy, 1997). It was later shown that plants possess two types of FARs, which differ in their subcellular localizations and preferred thioester-linked acyl substrate (acyl-CoA or acyl-ACP). Whereas the first type are classic microsomal enzymes residing in the endoplasmic reticulum (Rowland *et al.*, 2006), the second type are soluble plastid-localized proteins that use acyl-ACPs as substrates (Chen *et al.*, 2011; Shi *et al.*, 2011; Doan *et al.*, 2012). These differences have allowed for the engineering of WE production in plants in either seeds or the plastids of leaves (see below).

The protein structure of FARs displays two conserved domains, an N-terminal Rossmann-fold NAD(P)H binding domain, and a C-terminal fatty acyl-CoA reductase (FAR_C) domain. Their N-terminal extremity usually harbors the motif (I/Y/F)-L-(I/V)-(T/V)-G-X-X-T-G-F-L-A, a canonical ADP binding domain most probably involved NAD(P)H binding (Aarts *et al.*, 1997), and the classic YXXXK active site motif of short-chain dehydrogenase/reductase (Kavanagh *et al.*, 2008). Their C-term FAR_C domain is often annotated ‘sterile’ or ‘male sterile’ in databases because the Arabidopsis MALE STERILITY2 (MS2) protein was the first publicly available FAR sequence, but at that time with unknown function (Aarts *et al.*, 1997). FARs are usually about 500 amino acid polypeptides, with the plastidial isoforms containing an additional N-terminal targeting signal for chloroplast import (Chen *et al.*, 2011; Shi *et al.*, 2011; Doan *et al.*, 2012). In contrast, mammalian FARs contain an extra C-terminal transmembrane domain that allows for their anchoring to the peroxisomal membrane (Heilmann *et al.*, 2012). A similar hydrophobic region on the C-terminus was identified in AmFAR1 from honey bee (Teerawanichpan *et al.*, 2010), CfFAR2 and CfFAR3 from copepod *Calanus finmarchicus* (Teerawanichpan and Qiu, 2012), and avian FARs (Hellenbrand *et al.*, 2011).

Interestingly, FARs with both wide and strict substrate specificities have been described. Among bacterial FARs, two FARs from *Marinobacter aquaeolei* VT8 (referred to as Maqu_2220 and Maqu_2507) have been intensively studied biochemically. Both displayed a broad specificity producing *in vitro* C10 to C20 fatty alcohols, while Maqu_2220 was additionally shown to reduce fatty acyl-CoAs, fatty acyl-ACPs, and fatty aldehydes to corresponding fatty alcohols (Wahlen *et al.*, 2009; Hofvander *et al.*, 2011; Willis *et al.*, 2011; Liu *et al.*, 2013). Despite the apparent contrasting results obtained in different heterologous host systems (reviewed in Rowland and Domergue, 2012), biochemical studies have generally shown that plant FARs have distinct substrate specificities with clear substrate chain-length preferences. For example, Arabidopsis FAR1, FAR4, and FAR5 mostly use saturated C22:0, C20:0, and C18:0 as substrates, respectively (Domergue *et al.*, 2010). FAR3/CER4 seems to be specific for the production of saturated C24:0 to C28:0 fatty alcohols (Rowland *et al.*, 2006), but the recent characterization of CER17 showed that FAR3/CER4 is also active on ω6 monounsaturated fatty acids of similar chain length (Yang *et al.*, 2017). Whereas most Arabidopsis FARs preferentially use saturated substrates, the seed-localized jojoba *Sc*FAR mainly produces C20:1 and C22:1 fatty alcohols (Metz *et al.*, 2000; Miklaszewska and Banaś, 2016). Recently, two *Brassica napus* CER4 homologs were shown to be specific for reducing branched (anteiso) fatty acids (Liu *et al.*, 2021). Similarly, some insect FARs were shown to prefer monounsaturated substrates and to have a strong specificity toward the configuration (*cis*/*trans*) of the double bond (Lassance *et al.*, 2010). Altogether, these studies suggest that FARs with unique substrate specificities in terms of chain length, degree of saturation, and branching may exist in nature to produce tailored WEs. Nevertheless, although crystals were obtained from the plastidial FAR Defective in Pollen Wall (DPW) from rice (Wang *et al.*, 2014), no X-ray or NMR structure has yet been reported for any FAR, including non-plant FARs, hampering the understanding of substrate specificity at the amino acid level, and its modification for industrial applications.

### Wax synthases

WSs (EC 2.3.1.75) are acyltransferases, which catalyse esterification of an activated acyl chain with a fatty alcohol. The final step of WE formation has been investigated in different organisms since the 1960s (Kolattukudy, 1967; Wu *et al.*, 1981). Purification of the enzyme with WS activity from jojoba developing embryos permitted the cloning of the first WS coding sequence (Lardizabal *et al.*, 2000). The cDNA encoded a predicted protein of 352 amino acids with seven to nine transmembrane domains. The enzyme exhibited a rather broad substrate specificity in *in vitro* assays with microsomal membrane fractions. For the protein isolated from jojoba embryos, Lardizabal *et al.* (2000) reported a preference for 20:1-CoA, 18:1-OH, and 18:2-OH, while the enzyme present in microsomes isolated from yeast expressing *ScWS* gene exhibited highest activity towards 14:0-CoA, 16:0-CoA, and 20:1-OH (Miklaszewska and Banaś, 2016). Further studies led to the identification of WSs of different origins, which can be classified into three unrelated groups: bacterial-type bifunctional WS/acyl-CoA:diacylglycerol acyltransferase (DGAT) with additional TAG-synthesizing activity, jojoba (plant)-type WSs, and WSs from vertebrates, which are related to DGAT-type 2 proteins. Interestingly, the WS from jojoba shares an origin with DGAT-type 1 enzymes, whereas the Arabidopsis WS producing the WEs present in leaf epicuticular waxes (WSD1) is related to bacterial WS/DGAT (Yuan *et al.*, 2020).

The bacterial type WS/DGAT was first identified and characterized in *Acinetobacter baylyi* strain ADP1 (formerly *A. calcoaceticus*) (Kalscheuer and Steinbüchel, 2003). Homologous proteins were also found in members of *Mycobacterium*, *Rhodococcus*, *Streptomycetes*, and *Psychrobacter* genera (reviewed in Wältermann *et al.*, 2007; Röttig and Steinbüchel, 2013). WS/DGATs are composed of approximately 450–570 amino acids and due to their amphiphilic character they are not only associated with membranes or lipid inclusions, but also partially located in the cytosolic fraction (Stöveken *et al.*, 2005; Röttig and Steinbüchel, 2013). All WS/DGATs possess a highly conserved HHXXXDG motif, which is crucial for the acyltransferase activity (Wältermann *et al.*, 2007; Stöveken *et al.*, 2009; Villa *et al.*, 2014). Most studies have focused on WS/DGATs from *A. baylyi* (Kalscheuer *et al.*, 2003; Stöveken *et al.*, 2005), *Marinobacter aquaeolei* (Barney *et al.*, 2013; Vollheyde *et al.*, 2020), and *Marinobacter hydrocarbonoclasticus* (Holtzapple and Schmidt-Dannert, 2007; Miklaszewska *et al.*, 2018). Petronikolou and Nair (2018) reported the first crystal structure of *M. aquaeolei* WS/DGAT, which enabled the identification of the substrate-binding sites and engineering of enzyme specificity. Bacterial WS/DGATs have been shown to accept a wide range fatty acyl-CoAs and fatty alcohols, including cyclic, branched, and aromatic alcohols, with preference towards C14–C18 substrates (Stöveken *et al.*, 2005; Holtzapple and Schmidt-Dannert, 2007; Barney *et al.*, 2012; Shi *et al.*, 2012; Miklaszewska *et al.*, 2018). Members of the WS/DGAT family were also identified in plants, such as Arabidopsis (WSD1 to 11) (Li *et al.*, 2008), *Petunia hydrida* (PhWS1) (King *et al.*, 2007), sunflower (*Helianthus annuus*, HaWS) (Shalini and Martin, 2020), and oil palm (*Elaeis guineensis*; EgWS/DGAT_1–3) (Rosli *et al.*, 2018; Yuan *et al.*, 2020). WSD1, PhWS1, and EgWS/DGAT_1 possess two putative transmembrane domains and showed no or very low DGAT activity, which indicates that these enzymes are mainly involved in WE synthesis (King *et al.*, 2007; Li *et al.*, 2008; Rosli *et al.*, 2018; Yuan *et al.*, 2020). Recent identification and characterization of WS/DGATs from *E. gracilis* (Tomiyama *et al.*, 2017), the protist *Thraustochytrium roseum* (Zhang *et al.*, 2017), and the diatom *Phaeodactylum tricornutum* (Cui *et al.*, 2018) suggests that these bifunctional acyltransferases are more widespread than previously thought.

Jojoba-type WSs belong to a superfamily of membrane-bound *O*-acyltransferases and are related to DGAT-type 1 enzymes. They can be found in various plants, microalgae, and protists, but only a few of them, such as OsWS1 from rice (Xia *et al.*, 2015), EgWS from *E. gracilis* (Teerawanichpan and Qiu, 2010), and CzWS1 from *Chromochloris zofingiensis* (Xu *et al.*, 2021), were analysed in detail.

Among mammalian WSs, termed acyl-CoA:wax alcohol acyltransferases (AWATs), mainly mouse (*Mus musculus*) WS (MmWS or AWAT2) has been studied (Cheng and Russell, 2004*a*; Miklaszewska *et al.*, 2013; Kawelke and Feussner, 2015; Widjaja-Adhi *et al.*, 2020). The enzyme showed preference towards medium- and long-chain acyl-CoAs and fatty alcohols (Cheng and Russell, 2004*a*; Miklaszewska *et al.*, 2013). The specificity of mouse AWAT2 is determined by two neighboring transmembrane domains at the N-terminus of the protein (Kawelke and Feussner, 2015). Based on the similarity to mammalian AWATs, several avian WSs with diverse substrate specificities were identified in chicken (*Gallus gallus*), barn owl (*Tyto alba*) and goose (*Anser domesticus*). When tested in yeasts, GgWS1 from chicken produced the highest amount of WEs. GgWS1 and GgWS2 efficiently synthesized WEs from branched acyl-CoA and fatty alcohols, while AdWS4 from goose and TaWS4 from barn owl utilized isoprenols (Biester *et al.*, 2012a).

### FAR–WS fusion protein

In *Tetrahymena* species and related unicellular ciliate protozoa, FARs are often found fused to an acyltransferase domain. Dittrich-Domergue and coworkers (2014) showed that in *Tetrahymena thermophila* this bifunctional peroxisomal protein is involved in the early step of ether lipid biosynthesis: the N-terminal FAR domain produces a fatty alcohol, while the C-terminal domain generates *sn*-1-acyl-dihydroxyacetone phosphate. These two substrates are then used by a third enzyme, the alkyl-dihydroxyacetone phosphate synthase, to initiate ether lipid biosynthesis. The existence of a protein carrying both FAR and acyltransferase activity suggested that combining both FAR and WS domains in a single polypeptide may permit WE production by expressing a gene encoding a single bifunctional protein. Such a strategy indeed appeared successful for the engineering of WE production in plant seeds and leaves (see below).

## Oilseed platforms for wax ester production

The first successful production of WEs in a non-WE-storing oilseed plant was achieved using Arabidopsis (Lardizabal *et al.*, 2000). This proof-of-concept report initiated further studies on the possibility of producing WEs using oilseed crops such as *Camelina sativa*, *Crambe abyssinica*, *Brassica carinata*, and *Lepidium campestre*, most of which were carried out within the EU 7FP international project ICON (Industrial crops producing added value oils for novel chemicals; https://cordis.europa.eu/project/id/211400). These species were selected as oil crop platforms for WE production for several reasons (reviewed in Carlsson, 2009; Bansal and Durrett, 2016; Samarappuli *et al.*, 2020). Most importantly, they are cultivated only for industrial purposes, which minimizes the risk of admixing into the food oil crops (Carlsson, 2009). In addition, these crops have favorable agronomic properties, interesting seed fatty acid profiles, and can be grown on marginal land (Zhu *et al.*, 2016; Ivarson *et al.*, 2017).

### FAR and WS combinations and wax ester yields

To date, different attempts to engineer plants for WE production have been reported. The yields and composition of WEs accumulated in the host species harboring various combinations of WE-synthesizing genes are summarized in Table 1 (see Box 2 for FAR and WS nomenclature).

**Table 1.** Summary of modified plant species, enzyme combinations, wax ester yields, and composition

Plant species	Enzyme combinations used	Wax ester content^*a*^	Predominant wax esters	Reference
*A. thaliana*	MmFAR1 + MmWS	22 mg g^−1^ seed	20:1–18:2 (16 mol%) 18:1–18:1 (9 mol%) 20:0–18:1 (7 mol%)	Heilmann *et al.* (2012)
	Oleo3-MmFAR1 + Oleo3-MmWS	45 mg g^−1^ seed	20:1–18:2 (13 mol%) 18:1–18:2 (8 mol%) 20:0–18:2 (8 mol%)	
*A. thaliana*	MaFAR + ScWS	70 mg g^−1^ seed	18:1–20:1 (13.7 mol%) 20:1–20:1 (7.9 mol%) 18:1–16:0 (5.9 mol%)	Iven *et al.* (2013)
*A. thaliana*	Oleo3-MmFAR1 + Oleo3-MmWS	33 mg g^−1^ seed (17% of the oil^*b*^)	16:0–18:2 (14.5 mol%) 20:1–18:2 (11.5 mol%) 18:1–18:2 (11.3 mol%)	Iven *et al.* (2016)
	Oleo3-MmFAR1 + ScWS	21 mg g^−1^ seed (10% of the oil)	18:1–20:1 (22.6 mol%) 18:2–20:1 (14.5 mol%) 20:1–20:1 (10.0 mol%)	
	MaFAR + ScWS	108 mg g^−1^ seed (49% of the oil)	18:1–20:1 (17.7 mol%) 20:1–20:1 (10.5 mol%) 18:1–18:1 (10.1 mol%)	
*C. sativa*	Oleo3-MmFAR1 + Oleo3-MmWS	12 mg g^−1^ seed (6% of the oil)	16:0–18:2 (14.6 mol%) 18:0–18:2 (11.7 mol%) 18:1–18:2 (10.9 mol%)	Iven *et al.* (2016)
	Oleo3-MmFAR1 + ScWS	21 mg g^−1^ seed (9% of the oil)	18:1–20:1 (15.7 mol%) 18:3–20:1 (13.0 mol%) 18:2–20:1 (11.4 mol%)	
	MaFAR + ScWS	47 mg g^−1^ seed (21% of the oil)	18:1–20:1 (16.3 mol%) 20:1–20:1 (15.6 mol%) 18:0–20:1 (7.4 mol%)	
*A. thaliana*	MaFAR + ScWS	95 mg g^−1^ seed (41% of the oil)	WEs containing 18:1-OH (40 mol%), 20:1-OH (20 mol%), and 20:1-FA (38 mol%)	Yu *et al.* (2018)
	ScWS-MaFAR fusion protein +MaFAR	23 mg g^−1^ seed (13% of the oil) 64 mg g^−1^ seed (31% of the oil)	WEs containing 20:1-OH (45–52 mol%), 18:1-OH (20–28 mol%), and 20:1-FA (40 mol%)	
	MaFAR + TM-AbWS/DGAT	17 mg g^−1^ seed (7% of the oil)	WEs containing 18:1-OH (50 mol%), 18:2-OH (30 mol%) and 18:0-FA (40 mol%), 18:1-FA (30 mol%)	
	MaFAR + MaWS2	14 mg g^−1^ seed (6% of the oil)	WEs containing 18:1-OH (60 mol%), 18:2-OH (20 mol%) and 18:0-FA (60 mol%), 18:1-FA (20 mol%)	
	MaFAR + AbWS/DGAT	4 mg g^−1^ seed (3% of the oil)	20:1–18:1 (16 mol%) 18:1–18:1 (11 mol%) 20:1–20:1 (10 mol%)	
*A. thaliana*	MaFAR + MaWS2	22 mg g^−1^ seed (8% of the oil)	20:1–20:1 (12 mol%) 20:1–18:1 (9 mol%) 18:1–20:1 (6 mol%)	Vollheyde *et al.* (2021)
	tpMaFAR + tpMaWS2	12 mg g^−1^ seed (4% of the oil)	18:0–18:0 (16 mol%) 18:0–16:0 (14 mol%) 18:0–18:1 (7 mol%)	
	MaFAR + MaWS5	19 mg g^−1^ seed (7% of the oil)	20:1–18:1 (10 mol%) 20:1–20:1 (10 mol%) 20:1–16:0 (6 mol%)	
	tpMaFAR + tpMaWS5	12 mg g^−1^ seed (7% of the oil)	18:0–18:2 (9 mol%) 18:0–18:1 (7 mol%) 18:0–16:0 (7 mol%)	
*A. thaliana fae1 fad2*	Oleo3-MmFAR1 + Oleo3-MmWS	17–18 mg g^−1^ seed (8% of the oil)	18:1–18:1 (65 mol%) 16:0–18:1 (8 mol%)	Heilmann *et al.* (2012); Iven *et al.* (2016); Yu *et al.* (2018)
	Oleo3-MmFAR1 + ScWS	22 mg g^−1^ seed (16% of the oil)	18:1–18:1 (54 mol%) 18:1–16:0 (13 mol%)	
	MaFAR + ScWS	86 mg g^−1^ seed (42% of the oil)	18:1–18:1 (61 mol%) 18:1–16:0 (12 mol%)	
	MaFAR + AbWS/DGAT	5 mg g^−1^ seed	18:1–18:1 (62 mol%) 18:0–18:1 (9 mol%)	
*C. sativa* HO	MaFAR + ScWS	44 mg g^−1^ seed (20% of the oil)	18:1–18:1 (34 mol%) 18:1–16:0 (12 mol%) 18:1–20:1 (9 mol%)	Yu *et al.* (2018)
*C. sativa*	MaFAR + MhWS2	48.3 nmol/seed (29 mg g^−1^ seed)^*c*^	WEs containing C18 (47 mol%), C20 (28 mol%), C22 (10 mol%), C16 (6 mol%) acyl moieties, and C20 (61 mol%) and C18 (30 mol%) alcohol moieties	Ruiz-Lopez *et al.* (2017)
	MaFAR + MhWS2 + Thio10 MaFAR + MhWS2 + Thio12	43.5 nmol/seed (23 mg g^−1^ seed) 44.8 nmol/seed (23 mg g^−1^ seed)	WEs with slightly reduced C18–C24 acyl moieties and slightly increased ≤C14 and C16 acyl moieties compared with *Ma*FAR+*Mh*WS	
	MaFAR + MhWS2 + Thio14	34.0 nmol/seed (18 mg g^−1^ seed); 67.4 nmol/seed (42 mg g^−1^ seed) for T_3_	WEs containing C18 (34%), C20 (22%), C16 (18.6%) and ≤C14 (13.3%) acyl-moieties, and C20 (50 mol%), C18 (30 mol%) and C16 (16%) alcohol moieties	
	MaFAR + MmWS MaFAR + MmWS + Thio10 MaFAR + MmWS + Thio12 MaFAR + MmWS + Thio14	27.1 nmol/seed (10 mg g^−1^ seed) 74.6 nmol/seed (27 mg g^−1^ seed) 65.1 nmol/seed (31 mg g^−1^ seed) 33.5 nmol/seed (13 mg g^−1^ seed); 77.6 nmol/seed (32 mg g^−1^ seed) for T_3_	All MmWS combinations: WEs containing C18 (78–80 mol%), C20 (10–12 mol%), and C16 (5–8 mol%) acyl moieties, and C20 (60–70 mol%) and C18 (25–30 mol%) alcohol moieties	
*C. abyssinica*	ScFAR + ScWS	90 mg g^−1^ seed (23% of the oil) T_7_ generation—24.9% of the oil	22:1–20:1 (28 mol%) 22:1–22:1 (26 mol%) 22:1–18:2 (4 mol%)	Zhu *et al.* (2016); Li et al. (2019)
	ScFAR + ScWS + ScFAE1	55 mg g^−1^ seed (17% of the oil) T_7_ generation—18% of the oil	22:1–20:1 (18 mol%) 22:1–22:1 (9 mol%) 24:1–22:1 (6 mol%)	
*B. carrinata* HEA	ScFAR + ScWS	50 mg g^−1^ seed (24% of the oil)	22:1–22:1 (33 mol%) 22:1–20:1 (27 mol%) 22:1–18:1 (4 mol%)	Zhu *et al.* (2016)
	ScFAR + ScWS + ScFAE1	48 mg g^−1^ seed (24% of the oil)	24:1–24:1 (18 mol%) 24:1–22:1 (11 mol%) 22:1–24:1 (10 mol%)	
*C. sativa*	ScFAR + ScWS	48 mg g^−1^ seed (25% of the oil)	24:0–20:1 (12 mol%) 22:1–20:1 (11 mol%) 22:0–20:1 (10 mol%)	Zhu *et al.* (2016)
	ScFAR + ScWS + LaFAE1	52 mg g^−1^ seed (28% of the oil)	24:1–24:1 (21 mol%) 24:1–24:0 (20 mol%) 24:1–22:1 (11 mol%)	
	ScFAR + ScWS + LaFAE1 + *CsFAD2*-RNAi	60 mg g^−1^ seed (32% of the oil)	22:1–20:1 (25 mol%) 24:1–24:1 (13 mol%) 22:1–22:1 (11 mol%)	
*L. campestre*	ScFAR + ScWS	Up to 44.7 mg g^−1^ seed	22:1–20:1 (22.5 mol%) 22:1–22:1 (15.1 mol%) 22:1–18:1 (6 mol%)	Ivarson *et al.* (2017)
	ScFAR + ScWS + ScFAE1	Up to 85.8 mg g^−1^ seed	22:1–20:1 (16.2 mol%) 24:1–20:1 (9 mol%) 24:1–22:1 (8 mol%)	
*N. benthamiana* (transient)	tpMaFAR + AtPES2	1.62 nmol mg^−1^ leaf FW (0.9% leaf DW)	WEs containing 12:0 (45 mol%) and 14:0 (35 mol%) acyl moieties, and 16:0 (75 mol%) and 18:0 (25 mol%) alcohol moieties	Aslan *et al.* (2014)
	AtFAR6 + AtPES2	0.9 nmol mg^−1^ leaf FW	WEs containing 12:0 (45 mol%) and 14:0 (35 mol%) acyl moieties, and 16:0 (90 mol%) and 18:0 (10 mol%) alcohol moieties	
	tpMaFAR::MhWS	0.4 nmol mg^−1^ leaf FW	WEs containing 16:0 (55 mol%) and 18:0 (20 mol%) acyl moieties, and 16:0 (60 mol%) and 18:0 (35 mol%) alcohol moieties	
*N. benthamiana* (stable)	tpMaFAR::MhWS	0.28 µmol g^−1^ leaf FW or stem FW (0.15% DW)	WEs containing 16:0 (27 mol%), 18:0 (17 mol%), 20:0 (21%) and 22:0 (19%) acyl moieties, and 16:0 (55 mol%) and 18:0 (40 mol%) alcohol moieties	Aslan *et al.* (2015b)

^*a*^ For Iven *et al.* (2016) and Yu *et al.* (2018), the best performing transgenic lines were listed.

^*b*^ Relative WE content in the total seed oil.

^*c*^ Due to significant variations in seed weight, the authors emphasized that quantity of WE per seed better illustrated the enzyme activities than WE quantity per g of seeds (Ruiz-Lopez *et al.*, 2017); however, the latter values were included in the table for comparison with the other studies.

Abbreviations: AbWS/DGAT, bifunctional WS/DGAT from *Acinetobacter baylyi*; AtFAR6, fatty acyl reductase 6 from *Arabidopsis thaliana*; AtPES2, phytyl ester synthase 2 from *Arabidopsis thaliana*; *CsFAD2*-RNAi, RNAi construct for silencing *Camelina sativa* fatty acid desaturase 2 gene; DW, dry weight; FW, fresh weight; HEA, high erucic acid; HO, high oleate; LaFAE1, 3-ketoacyl-CoA synthase from *Lunaria annua*; MaFAR, fatty acyl reductase from *Marinobacter aquaeolei*; MaWS2, wax synthase 2 from *Marinobacter aquaeolei*; MaWS5, wax synthase 5 from *Marinobacter aquaeolei*; MhWS2, wax synthase 2 from *Marinobacter hydrocarbonoclasticus*; Oleo3-MmFAR1, fatty acyl reductase 1 from *Mus musculus* fused with Oleo3 and lacking peroxisome-targeting C-terminal sequence; Oleo3-MmWS, wax synthase from *Mus musculus* fused with Oleo3; ScFAR, fatty acyl reductase from *Simmondsia chinensis*; ScFAE1, 3-ketoacyl-CoA synthase from *Simmondsia chinensis*; ScWS, wax synthase for *Simmondsia chinensis*; Thio10, 10:0-ACP thioesterase from *Cuphea hookeriana*; Thio12, 12:0-ACP thioesterase from *Umbellularia californica*; Thio14, 14:0-ACP thioesterase from *Cuphea palustris*; TM-AbWS/DGAT, bifunctional WS/DGAT from *Acinetobacter baylyi* fused with two transmembrane domains from MmWS at the N-terminus; tpMaFAR, MaFAR fused with a transit peptide from AtFAR6 (Aslan *et al.*, 2014) or a transit peptide (80 amino acid residues) of the small subunit of rubisco complex (Vollheyde *et al.*, 2021); tpMaFAR::MhWS, fusion of tpMaFAR and MhWS2; tpMaWS, MaWS fused with a transit peptide (80 amino acid residues) of the small subunit of rubisco complex.

First studies focused on maximizing WE yields and tailoring WE composition by employing various combinations of FARs and WSs of different origin. The mouse FAR (MmFAR1) and WS (MmWS), which showed preference for 16–18C saturated and unsaturated substrates (Cheng and Russell, 2004a, b), were used for the synthesis of WEs composed of long carbon chains with a maximum of one double bond per alcohol and acid moiety, which are highly suitable for industrial applications (Heilmann *et al.*, 2012). Since MmFAR1 and MmWS localize to different organelles (peroxisomes and the endoplasmic reticulum, respectively), a C-terminal peroxisomal targeting signal was removed from MmFAR1, and both enzymes were fused with Arabidopsis oleosin 3, a lipid droplet protein. Both genes were expressed under the seed-specific napin promoter. Co-targeting MmFAR and MmWS to lipid droplets resulted in 2-fold increase in WE accumulation in Arabidopsis seeds compared with the unmodified enzymes (from 22 to 45 mg g^−1^ seed), without affecting the WE composition. In both cases, the most abundant WE species were gondoyl linoleate (20:1–18:2), oleyl linoleate (18:1–18:2) and arachidyl linoleate (20:0–18:2) (Heilmann *et al.*, 2012). A further increase in WE yield was obtained (70 mg g^−1^ seed) when the jojoba WS (ScWS) was used in combination with the major FAR providing fatty alcohols for WE production in *M. aquaeolei* (MaFAR, Maqu_2220; Hofvander *et al.*, 2011). Accumulated WEs were mainly composed of 18:1 and 20:1 alcohol moieties and 20:1 and 18:1 acyl moieties, with a predominance of oleyl gondoate (18:1–20:1) (Iven *et al.*, 2013).

The efficiency of different combinations of MmFAR, MaFAR, MmWS, and ScWS in producing WEs was then tested in Arabidopsis and *C. sativa* seeds (Iven *et al.*, 2016). The expression of *MaFAR* and *ScWS* genes resulted in the highest levels of WEs, reaching 108 mg g^−1^ seed in Arabidopsis and 47 mg g^−1^ seed in *C. sativa*. In contrast, transgenic lines with oleosin-fused MmFAR combined with MmWS or ScWS produced considerably less WEs (up to 33 mg g^−1^ in Arabidopsis and up to 21 mg g^−1^ seed in *C. sativa*), which suggested that a low efficiency in fatty alcohol formation limited WE synthesis. The overall composition of accumulated WEs in Arabidopsis and *C. sativa* was similar, but the WE profiles were partially influenced by the substrate specificity of the utilized enzymes. For example, combinations with ScWS incorporated mainly 20:1, whereas WEs produced by MmWS contained mainly unsaturated C18 acyl moieties (Iven *et al.*, 2016).

Another study compared the activities of MmWS and MhWS2 from *M. hydrocarbonoclasticus* (MhWS2, corresponding to WS2 in Holtzapple and Schmidt-Dannert, 2007) in *C. sativa* (each in combination with MaFAR). MhWS2 was found to be more efficient, producing 48.3 nmol WE/seed (approximately 29 mg g^−1^ seed) compared with 27.1 nmol WE/seed produced by MmWS (Ruiz-Lopez *et al.*, 2017). Nevertheless, the use of other bacterial WSs did not improve yield obtained by ScWS. For example, WE amounts accumulated in transgenic Arabidopsis lines, transformed with MaFAR together with WS1 from *M. aquaeolei* or the bifunctional WS/DGAT from *A. baylyi* containing two additional transmembrane domains from the mouse AWAT2, did not exceed 17 mg g^−1^ seed. The authors speculated that the preference of bacterial enzymes towards acyl-ACPs instead of acyl-CoAs might affect WE yields (Yu *et al.*, 2018).

Yu *et al.* (2018) also investigated whether FAR–WS fusion proteins can be used for WE production in Arabidopsis seeds. Transgenic lines with the ScWS–MaFAR fusion protein accumulated 23 mg g^−1^ seed of WEs, which was four times less compared with the MaFAR and ScWS combination (95 mg g^−1^ seed). However, these yields are not fully comparable because the gene encoding the fusion protein was under the control of β-conglycinin promoter, while the expression of the individual genes was driven by the napin promoter. Nevertheless, co-expressing the fusion protein gene and *MaFAR* led to an increase in WE amounts to 64 mg g^−1^ seed, which may suggest that the FAR, rather than the WS, activity of the fusion protein is limiting WE synthesis. Interestingly, ScWS–MaFAR fusion protein displayed a slightly different substrate specificity than the combination of individual enzymes, and preferentially incorporated 20:1-OH instead of 18:1-OH (Yu *et al.*, 2018).

These studies clearly showed that the employed FARs and WSs displayed different efficiencies in fatty alcohol and WE synthesis in transgenic seeds (Table 1). As a result, using different enzyme combinations impacted WE yields to a greater extent than the WE composition. Since only a limited number of the WE-synthesizing enzymes have been tested in plants, it would be interesting to investigate if other, not yet tested combinations of FARs and WS can produce higher amounts of WEs.

### Tailoring wax ester composition in seeds

**Fig. 3.**
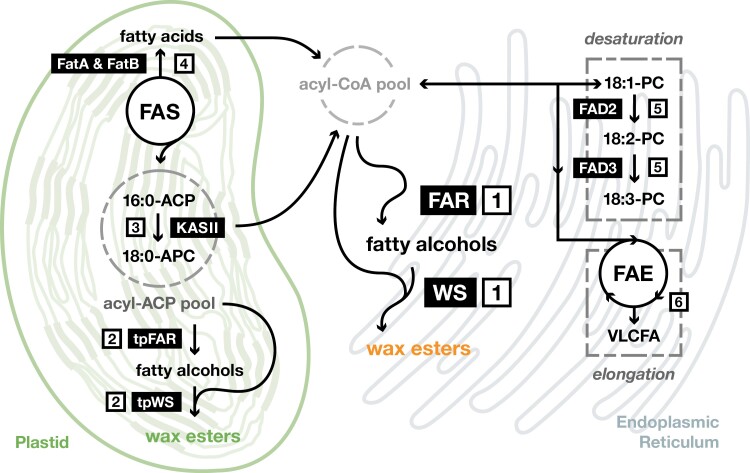
Schematic representation of target pathways for metabolic engineering of wax ester composition. Production of wax esters (WEs) can be engineered in the endoplasmic reticulum [1], or in the plastids by using a chloroplast-localized fatty acyl reductase (tpFAR) and wax synthase (tpWS) or non-plastidial enzymes fused with a transit peptide (tp) [2]. In both cases, selecting FARs and WSs with appropriate substrate specificities (or modifying these) can result in incorporation of the desired fatty acids and alcohols into the final WEs. The composition of the produced WEs can also be tailored by modifying the acyl-ACP [3] or acyl-CoA [4–6] pools, which contain FAR and WS substrates. Knocking out or down-regulating *KASII* gene, encoding the plastidial enzyme converting 16:0-ACP to 18:0-ACP, increases the 16:0 level in the acyl-ACP pool [3]. Introducing C10 to C14-specific acyl-ACP thioesterases (FatA and FatB), which prematurely terminate plastidial fatty acid synthesis, increases the levels of medium chain fatty acids in the acyl-CoA pool [4]. Higher 18:1-CoA content can be achieved by knocking out or down-regulating genes encoding fatty acid desaturases FAD2 and FAD3 responsible for desaturating 18:1 and 18:2, respectively [5]. Co-expressing a gene encoding the β-ketoacyl-CoA-synthase from the fatty acid elongase complex (FAE) increases the content of very long-chain fatty acyl-CoA (VLCFAs, ≥C20) [6].

Several strategies were developed in parallel not to increase the WE yields, but for producing tailored WEs by modifying the acyl-chain pools used as substrates by FARs and WEs. Approaches targeting either fatty acid synthesis in plastids or different pathways involving endoplasmic reticulum-localized enzymes were tested (Fig. 3).

In order to increase the levels of medium-chain WEs, C10 to C14-specific acyl-ACP thioesterases from *Cuphea* and *Umbellularia* species, which prematurely terminate plastidial fatty acid synthesis, were used by Ruiz-Lopez *et al.* (2017). The genes encoding these thioesterases were co-expressed in *C. sativa* seeds together with genes encoding FARs and WSs from mouse (*MmWS*), *M. aquaeolei* (*MaFAR*, Maqu_2220), and *M. hydrocarbonoclasticus* (*MhWS2*). Additional thioesterase activity resulted in reducing the content of C36–C38 species and accumulating C30–C34 for the combinations of *MaFAR* and *MhWS2*, especially for the 14:0-ACP thioesterase. Only a small effect on WE composition was observed in the case of *MmWS*-containing constructs despite considerable changes in the acyl-CoA pool in the transgenic lines. Interestingly, introduction of thioesterases not only changed the WE profiles, but also increased WE levels for some of the tested combinations. The highest WE accumulation in T_2_ seeds was observed in lines with MaFAR, MmWS and 10:0-ACP thioesterase (74.5 nmol WE/seed) and MaFAR, MhWS2 and 12:0-ACP thioesterase (44.8 nmol WE/seed). In T_3_ lines, further increases in WE levels were observed (Ruiz-Lopez *et al.*, 2017). Recently, Vollheyde *et al.* (2021) showed that targeting WE-synthesizing enzymes from *M. aquaeolei* to Arabidopsis plastids by fusion with a plastidial transit peptide resulted in accumulation of higher amounts of WEs containing saturated C16 and C18 moieties. Such a strategy can be useful for production of long-chain WEs without a need to overexpress genes involved in acyl-CoA pool modifications (Vollheyde *et al.*, 2021).

To enrich WEs in oleyl oleate (18:1–18:1), the 18:1-CoA content of the acyl-CoA pool was increased by knocking out or down-regulating Arabidopsis and *C. sativa* desaturase genes responsible for desaturating (FAD2 and FAD3) or elongating (FAE1) oleic acid (18:1) (Heilmann *et al.*, 2012; Iven *et al.*, 2016; Yu *et al.*, 2018). When oleosin-fused MmFAR and MmWS were introduced into Arabidopsis *fae1 fad2* mutant, which accumulates 85% of oleic acid in its seeds, the content of 18:1–18:1 reached 65% of seed WEs (only 5% in the wild type background), but also slightly decreased the yield (Heilmann *et al.*, 2012). Similar 18:1–18:1 contents in Arabidopsis *fae1 fad2* mutant were also obtained for co-expression of *ScWS* and *MmWS* (54–65 mol%, Iven *et al.*, 2016), and *MaFAR* and *AbWS/DGAT* (62 mol%, Yu *et al.*, 2018). For *C. sativa*, increased 18:1–18:1 accumulation (27–34 mol%) was achieved by crossing the *MaFAR/ScWS*-expressing line with the high oleate *C. sativa* line, generated by the RNAi approach to silence *FAD2*, *FAD3*, and *FAE1* (Yu *et al.*, 2018).

Accumulation of WEs enriched in very long acyl-chains was first attempted in Arabidopsis using the FAR and WS from jojoba together with a 3-ketoacyl-CoA synthase (KCS or FAE1, a component of the fatty acid elongase complex) from *Lunaria annua* to increase very long-chain fatty acid content in the acyl-CoA pool (Lardizabal *et al.*, 2000). A similar approach was later used to establish jojoba-like WE production in *C. abyssinica*, *C. sativa*, high erucic acid *B. carinata* (with *FAD2*-RNAi suppression and *C. abyssinica FAE1* overexpression) and *L. campestre* (Zhu *et al.*, 2016; Ivarson *et al.*, 2017; Li *et al.*, 2019). WEs accumulated in *C. abyssinica*, *C. sativa*, and *B. carinata*, co-expressing *ScFAR* and *ScWS*, accounted for approximately 25% of the total oil content. Individual *C. abyssinica* seeds containing more than 50% of WEs were also identified. WEs accumulated in *C. abyssinica* and *B. carinata* were mainly composed of 20:1-FA, 22:1-FA and 22:1-OH, whereas most abundant WEs accumulated in *C. sativa* consisted of 20:1-FA, 24:0-FA and C22–C24 saturated and unsaturated fatty alcohols. Further modifications of the WE composition were achieved by addition of *FAE1* gene from jojoba (*ScFAE1*) or *L. annua* (*LaFAE1*) and *FAD2*-RNAi suppression, which resulted in increased content of WEs with C24 acyl and alcohol moieties, and decrease in saturated and polyunsaturated components (Zhu *et al.*, 2016). Through direct selection based on WE content, *C. abyssinica* lines expressing *ScFAR*/*ScWS*, *ScFAR*/*ScWS*/*ScFAE1* and *ScFAR*/*ScWS*/*CaFAD2*-RNAi combinations with highly stabilized WE levels were further developed (Li *et al.*, 2019). Detailed analysis of the seed oil of T_7_ generations revealed that the average WE content in these lines was 25%, 18%, and 29%, respectively (Li *et al.*, 2019).

Studies on *L. campestre* demonstrated that wild species have a high potential as new industrial crops for engineered WE production. Lines harboring *ScFAR* and *ScWS* genes accumulated mainly C42 and C44 WEs with levels reaching 44.7 mg g^−1^ seed. Addition of *ScFAE1* led to higher WE amounts (up to 86 mg g^−1^ seed) with increased content of C46 and C48 species. Interestingly, these C46 and C48 WEs were accumulated in the seed coat rather than in the embryo, whereas other species, such as 42:2 or 44:2 were detected both in the embryo and the seed coat (Ivarson *et al.*, 2017).

Altogether, these results suggest that modifying the acyl-CoA pool composition can tremendously affect the final WE composition, but at the same time, its impact on the WE yields can vary from negative to positive (Table 1). In addition, it seems that reconstituting WE synthesis in mutant backgrounds or by down-regulating competing activities had a stronger effect on the WE composition than overexpressing new activities like thioesterases.

### Modifying FAR and WS specificities

Based on to the WE profiles obtained in different plant species and backgrounds with the various gene combinations, it can be concluded that WE composition is mainly influenced by the availability of acyl-CoAs, and to a lesser extent by the substrate specificity of FARs and WSs. However, matching the enzymes’ activity to the available substrates increases the likelihood of their efficient incorporation into WEs. The rapidly growing number of sequenced genomes has enabled the identification and characterization of putative FARs and WSs from different organisms. The extensive available data on their properties, substrate specificity, and activity in different expression systems provide a useful toolbox for designing the production of tailored WEs. Additionally, several studies showed that the substrate specificity of FARs and WSs can be engineered. Arabidopsis FAR5 and FAR8 have high (85%) amino acid sequence similarity but possess distinct substrate specificities towards 18:0-CoA and 16:0-CoA, respectively. Domain-swaps and site specific mutations revealed that two amino acid substitution (A355L and V377M) in FAR5 sequence changed enzyme specificity from 18:0-CoA to 16:0-CoA, whereas reciprocal substitution in FAR8 had the opposite effect (Chacón *et al.*, 2013). Different amino acid substitutions also enabled alteration of substrate specificity of bacterial WS/DGATs. Selection of residue potentially affecting the substrate specificity of *M. aquaeolei* WS/DGAT (Ma1) was based on sequence comparison with PapA5 acyltransferase from *Mycobacterium tuberculosis*. It was shown that specific changes in residues at positions 360, 356, and 405 can shift Ma1 enzyme selectivity for short-chain, medium-chain, branched, and aromatic fatty alcohols (Barney et al., 2013, 2015). Further residue substitutions, identified on the basis of the crystal structure of *M. aquaeolei* WS/DGAT, were introduced within the acyl-CoA-binding pocket and led to increased preference towards shorter acyl-CoAs (Petronikolou and Nair, 2018). Enzyme selectivity can also be modified by fusion with the domains determining substrate specificity, which was shown for the mouse AWAT2 carrying segments of mouse DGAT2 (Kawelke and Feussner, 2015), and for *A. baylyi* WS/DGAT fused with two transmembrane domains from mouse AWAT2 (Yu *et al.*, 2018).

To conclude, further development in metabolic engineering of tailored WE production in plants will most likely focus on combining efficient strategies to modify the acyl-CoA and/or acyl-ACP pools with suitable FARs and WSs.

### Limitations for wax ester accumulation in seeds

Studies on WE accumulation in different oilseed crops demonstrated that high WE contents negatively impacted seed germination. *C. sativa MaFAR/ScWS*-expressing lines with high WE amounts had white cotyledons and their germination was delayed (Iven *et al.*, 2016; Yu *et al.*, 2018). In the case of *C. abyssinica*, a decreased frequency of seed germination, reduced growth, and brown spots on the cotyledons were observed for seeds containing over 35% WEs in their oil, especially for the lines harboring the *ScFAR*/*ScWS*/*ScFAE1* combination (Li *et al.*, 2019). In field and greenhouse trials, *C. abyssinica* lines expressing *ScFAR*, *ScWS*, and *ScFAE1* had reduced seed yields and oil contents, and showed lower germination rates as well as delayed flowering and seed maturation. Similar effects were observed for *C. sativa ScFAR*/*ScWS*/*LaFAE1*-expressing lines grown in a greenhouse (Zhu *et al.*, 2016). Hampered germination was also reported for *L. campestre* seeds with high WE content. Interestingly, the embryos of the transgenic lines displayed disrupted neutral lipid packaging (Ivarson *et al.*, 2017).

The results above strongly suggest that the impaired seed germination and seedling establishment represent a bottleneck in accumulating very high WE levels in the seeds of oilseed crops. The lowest seed germination rates and seed yields were observed for transgenic *C. abyssinica* and *C. sativa* lines harboring additionally *ScFAE1* or *LaFAE1* gene. Since this additional expression resulted in higher amounts of WEs composed of C24 acyl and alcohol moieties, it was postulated that the production of very long-chain fatty acids and alcohols may have a negative impact on lipid metabolism in developing and germinating seeds (Zhu *et al.*, 2016; Li *et al.*, 2019). The accumulation of potentially toxic fatty alcohols and/or the inability of the host seed enzymatic machinery to metabolize WEs may be the origin of the observed impaired germination and seedling growth. In jojoba, the WE mobilization pathway relies on the activity of three enzymes. First, a lipase (wax ester hydrolase) hydrolyses WEs to fatty acids and fatty alcohols. Then, before entering β-oxidation, the free fatty alcohols need to be converted to the corresponding fatty acids, which is catalysed by a fatty alcohol oxidase and a fatty aldehyde dehydrogenase (Rajangam *et al.*, 2013). Since jojoba lipases exhibited high activity towards both WEs and TAGs (Kawiński *et al.*, 2021), it cannot be ruled out that lipases from oilseed crops have the ability to hydrolyse WEs. Therefore, introduction of the jojoba fatty alcohol oxidation pathway to WE-storing crops could promote WE degradation and thus improve seed germination. Finally, disturbances in seed yield, germination, and seedling establishment may also be a result of improper packaging of WEs into lipid droplets, which was observed for *L. campestre* (Ivarson *et al.*, 2017). The recently released complete jojoba genome (Sturtevant *et al.*, 2020) represents an excellent resource to better understand how this unique plant optimally packs and stores WE in its seeds, and efficiently degrades them for germination.

### Extraction and properties of wax esters accumulated in oilseeds

To compete with synthetic WEs, plant-based WEs need to have favorable properties for industrial applications, and methods for their extraction from seeds should be simple and cheap. The oil from WE-accumulating seeds can be recovered using standard solvent extraction preceded by seed crushing or pressing (Ruiz-Lopez *et al.*, 2017; Shirani *et al.*, 2020). The crucial step is the separation of WEs from TAGs. For WEs with melting points higher than TAGs, it is possible to use a process called winterization, which consists of gradual cooling of the oil resulting in crystallization of WEs (Zhu *et al.*, 2016). However, this method is not efficient enough for shorter WEs, which are characterized by lower melting points. Ruiz-Lopez *et al.* (2017) tested the efficiency of molecular distillation on a pilot-scale using 1200 *C. sativa* plants accumulating mixtures of C30–C40 WEs in the seeds (up to 30 mg g^−1^ seed). The procedure allowed recovery of over 80% of WEs from refined oil, whereas only 20% of WEs were extracted by winterization (Ruiz-Lopez *et al.*, 2017). Transgenic *C. abyssinica* plants containing 20% of WEs in their oil were used for development of another method of WE separation, which included a mild methylation step followed by short-path distillation. Addition of the purified wax esters to conventional crambe oil to the concentration of 15 wt% improved its temperature stability, oxidative resistance, wear resistance and lubrication properties at elevated temperatures (Shirani *et al.*, 2020). These findings demonstrate that WEs accumulated in seeds of transgenic oil crops can be extracted using rather simple procedures, and used for the production of added-value lubricants.

## Wax ester production in leaves

Whereas the worldwide demand for vegetable oil is increasing yearly, the arable land available for growing oilseed crops remains limited. In that context, producing lipids of interest in the vegetative tissues of high biomass crops has been proposed as a promising alternative (Carlsson *et al.*, 2011; Mitchell *et al.*, 2020). WE production in green tissues was first assayed by transiently co-expressing FAR and WS genes in *Nicotiana benthamiana* leaves. Aslan *et al.* (2014) tested several combination of two different FARs together with two WSs: AtFAR6 from Arabidopsis (Doan *et al.*, 2012) or MaFAR from *M. aquaeolei* (Maqu_2220) together with AtPES2 from Arabidopsis (Lippold *et al.*, 2012) or MhWS2 from *M. hydrocarbonoclasticus*. In order to address the enzymes of prokaryotic origin to the chloroplast, the authors fused the transit peptide sequence of AtFAR6 at their N-terminus (yielding tpMaFAR and tpMhWS).

The highest WE amount (1.62 nmol mg^−1^ FW, corresponding to approximately 0.9% of leaf DW, 5 days post-infiltration) was achieved using tpMaFAR in combination with AtPES2. Expressing *AtFAR6* resulted in WEs mainly composed of 16:0-OH, while combinations using tpMaFAR produced WEs containing both 16:0-OH and 18:0-OH. At the fatty acid level, WEs produced upon *tpMhWS* expression mainly contained 16:0 and 18:0, while expressing *AtPES2* resulted in WEs mainly comprising medium chain (12:0 and 14:0) fatty acids. Transition electron microscopy analyses indicated that WEs accumulated in the chloroplasts as aggregates of various shapes. The authors also tested the activity of a fusion polypeptide harboring the catalytic parts of both MaFAR and MhWS. Expression of *tpMaFAR::MhWS* resulted in WE yields in the same range as when expressing *tpMaFAR* and *MhWS* as separate polypeptides. Co-expression of Arabidopsis *WRINKLED1* gene (*AtWRI1*), a master positive regulator of fatty acid biosynthesis (Focks and Benning, 1998), generally did not increase WE yields, even though higher accumulation of TAGs, suggesting increased fatty acid production, was observed. Altogether, this first study showed that it is possible to divert *de novo* fatty acid biosynthesis in the chloroplast to WE synthesis, and that using enzymes differing in their substrate preferences may permit the production of specific mixtures of WEs.

In a following study, Aslan *et al.* (2015a) attempted to additionally inhibit *KASII* expression using RNAi technology in leaves of *N. benthamiana*. KASII is involved in C16 to C18 fatty acid conversion within the plastid. Although transient RNAi approaches led to an almost complete inhibition of *KASII* expression, only a moderate increase in WE production was observed for enzyme combinations containing AtFAR6 (from 0.94 to 1.63 nmol mg^−1^ FW). Similarly, the 16 to 18 ratio was increased upon expression of *AtFAR6*, but not when *tpMaFAR* was used. In agreement with a previous study showing that strong seed-specific hairpin-RNAi reduction of *KASII* expression led to lethality (Pidkowich *et al.*, 2007), leaves agro-infiltrated with the *KASII*-RNAi construct displayed bleaching symptoms.

Finally, Aslan *et al.* (2015b) tackled WE production in green tissues, not using transient expression, but stably transforming tobacco plants. In this study, they expressed a gene encoding a fusion protein between two bacterial enzymes (*tpMaFAR::MhWS*) under the control of the cauliflower mosaic virus 35S promoter in *N. benthamiana.* The best transgenic plants obtained showed an 8-fold increase in WEs at the whole plant level, reaching 0.28 µmol g^−1^ FW in leaves. WEs were also detected in stems, predominantly in the middle part. As expected from previous works, the produced WEs were mainly composed of C16 and C18 fatty acids and fatty alcohols. Nevertheless, the detection of very long-chain fatty acids (20:0 and 22:0) in the WE fatty acid fraction suggests that although the fusion polypeptide was targeted to the plastids, some of the WE production might have occurred outside. The authors also showed that the chlorotic leaf phenotype observed in some lines producing WEs was most likely caused by accumulation of free fatty alcohols. This suggests that future strategies should use highly active WSs to efficiently convert all the fatty alcohols produced into WEs, thus avoiding negative effects on plant health and increasing WE yields.

## Conclusions and future prospects

The past two decades have witnessed impressive progress in the metabolic engineering of WE production in plants. Pioneering studies successfully demonstrated the feasibility of WE accumulation in seeds of TAG-storing crops, such as *C. sativa*, *C. abyssinica*, *B. carinata*, and *L. campestre*. Additionally, using a wide range of WE-synthesizing enzymes with desired specificities together with different approaches to modulate the acyl-CoA pool enabled the specific accumulation of certain WE molecular species. However, the strategies developed so far have only yielded viable transgenic crops with limited levels of WEs. Therefore, further research should focus on increasing WE accumulation by identifying and overcoming possible bottlenecks (see Box 3). Lipidomics, metabolomics, and visualization of the WE spatial distribution *in situ* using MALDI-MSI will certainly advance our understanding of implementing WE metabolism in transgenic seeds, and unravel underlying limitations. Localizing both FAR and WS enzymes to the same subcellular compartment (or even subdomains of compartments) to increase their physical proximity, using single enzymes or fusion proteins, might improve substrate channeling and thus WE yields. The competitiveness of plant-based WE production could also be increased by establishing the synthesis of WEs with even higher lubrication properties, such as WEs with hydroxy groups or branched acyl-chains. Such WEs have already been described in nature, and some FAR and WS enzymes with suitable specificities have been identified in birds, plants, and protists. Nevertheless, their potential, as well as that of many so far uncharacterized enzymes, for producing tailored WE in plants still needs to be evaluated. In addition, molecular modeling and directed evolution approaches may allow optimization of the substrate specificity of certain FAR and WS enzymes. Most importantly, a better understanding of why seeds with high WE contents are impaired in germination is essential for generating valuable transgenic plant lines stable over multiple generations. Genes allowing normal germination despite high levels of seed WEs must be present within the recently published jojoba genome. Another strategy to be tested is to produce WEs in the vegetative tissues of high biomass crops such as sorghum or sugarcane, optimally during senescence when thylakoid membranes and lipids are remobilized. To conclude, even if the first attempts to modify plant lipid metabolism towards the production of WEs have been promising, a lot more still needs to be achieved in order to establish sustainable sources of bio-lubricants in plants.

**Box 1. Wax ester nomenclature**

The names of WEs are formed in the same way as the names of other esters. The first part of the name specifies the alkyl residue derived from the alcohol (alcohol residue), and the second part is derived from the acid residue. In literature, both common and IUPAC fatty acid and fatty alcohol names are used. The shorthand notation used in different reports can cause some confusion. Usually, it reflects the WE name: the alcohol residue precedes the acyl residue and they are connected with a hyphen. In the alternative shorthand notation the acyl residue is given first, but then a slash is used instead of a hyphen (Chen *et al.*, 2015). However, in some studies these two notations were mixed: in the traditional notation a hyphen was replaced by a slash (e.g. 20:0/18:2 was used for arachidyl linoleate). Examples of WE names with the shorthand notation format used in this review are given below.

**Table:** 

**WE common name**	**Alcohol residue**	**Acyl residue**	**Shorthand notation**
Lauryl stearate	12:0-OH	18:0	12:0–18:0
Cetyl/palmityl gondoate	16:0-OH	20:1^a^	16:0–20:1
Linoleyl arachidate	18:2-OH	20:0	18:2–20:0
Linolenyl oleate	18:3-OH	18:1	18:3–18:1

a XX:Y indicates a fatty acyl chain with XX carbons and Y unsaturation. Like other lipids, WEs can be classified according to the length of their acyl-chains. Most common WEs are long-chain WEs, which are composed of C16 and C18 acyl moieties, and very long-chain WEs with >C18 acyl chains. WE containing <C8 and C8–C14 acyl-chains are referred as to short and medium, respectively.

**Box 2. Nomenclature of enzymes used for wax ester synthesis in plants**

Since various combinations of enzymes derived from different organisms have been used for WE production in plants, a unified nomenclature is important for data comparison. Most of the abbreviations used in the papers discussed in this review follow a two-letter prefix convention with the first letter uppercase and the second letter lowercase (for example, MaFAR—fatty acyl reductase from *Marinobacter aquaeoli*; ScWS—wax synthase from *Simmondsia chinensis*). Therefore, we adopted this convention here. However, it should be noted that some studies used a different nomenclature to facilitate the analysis of the results (for example in Ruiz-Lopez *et al.*, 2017). Some discrepancies in the literature may also occur in the case of mammalian and bacterial WE-synthesizing enzyme. Although in 2005 it was proposed to use the name acyl-CoA wax alcohol acyltransferase (AWAT) for mammalian wax synthases (Turkish *et al.*, 2005), both abbreviations MmWS and AWAT2 frequently appear for the mouse WS. The variations in abbreviations used for bacterial wax synthases (WS, WS/DGAT, WSD) result from bifunctionality of these enzymes. In addition, for some enzymes, other names are commonly used (for example, AtfA for WS/DGAT from *Acinetobacter baylyi* or Maqu_2220 for one of the FARs from *Marinobacter aquaeolei*).

**Box 3. Current and future challenges in further development of wax ester production in plants**

- Identifying and overcoming the reasons for impaired germination and seedling growth of transgenic plant lines with high WE content.
- Improving WE packaging in seeds.
- Increasing WE levels in seed oil.
- Engineering synthesis of WE blends with defined composition suited for industrial applications.
- Establishing production of unusual WEs with outstanding lubrication properties.
- Further optimization of WE production in vegetative tissues.

## Abbreviations

ACP: acyl carrier protein
CoA: coenzyme A
DGAT: acyl-CoA:diacylglycerol acyltransferase
FAD: fatty acid desaturase
FAE: fatty acid elongase
FAR: fatty acyl reductase
TAG: triacylglycerol
WE: wax ester
WS: wax synthase
